# Clinical and demographic predictors of the need for pharmacotherapy in neonatal abstinence syndrome

**DOI:** 10.3389/fped.2025.1527276

**Published:** 2025-08-11

**Authors:** Shawana Bibi, Rachana Singh, Janis L. Breeze, Jason Nelson, Walter K. Kraft, Jonathan M. Davis

**Affiliations:** ^1^ Tufts Clinical and Translational Science Institute, Boston, MA, United States; ^2^ Cleveland Clinic Children’s Hospital, Case Western Reserve University Lerner College of Medicine, Cleveland, OH, United States; ^3^ Department of Pediatrics, Tufts University School of Medicine, Boston, MA, United States; ^4^ Department of Pharmacology and Experimental Therapeutics, Thomas Jefferson University, Philadelphia, PA, United States

**Keywords:** neonatal, abstinence syndrome, predictors, clinical, pharmacotherapy

## Abstract

**Objective:**

Development and validation of a clinical prediction model for receipt of pharmacotherapy for Neonatal Abstinence Syndrome (NAS).

**Study design:**

Data from three cohorts included *in- utero* opioid exposed neonates ≥37 weeks gestation. Primary outcome was the receipt of pharmacotherapy utilizing a modified Finnegan Neonatal Abstinence Scoring System (FNASS). A stepwise multivariable logistic regression model was built and internally validated.

**Results:**

Of 698 infants included, 430 received pharmacotherapy. The final model included seven predictors of receipt of pharmacotherapy: gestational age, exposure to maternal breast milk, type of maternal opioid medication, and exposure to heroin, cocaine, benzodiazepines, and/or antipsychotic medications. The model had an AUROC of 0.68 (95% CI: 0.64–0.72; optimism corrected 0.65).

**Conclusion:**

Our prediction model was parsimonious and identified seven predictors associated with the need for PT. Larger cohort studies are needed to more definitively establish risk of significant NAS requiring pharmacotherapy.

## Introduction

Neonatal Abstinence Syndrome (NAS) is characterized by signs of withdrawal that affects neonates following chronic exposure to opioids *in-utero*, often with co-exposure to other psychotropic substances. There has been an exponential rise in Opioid Use Disorder (OUD) in pregnancy over the past two decades, resulting in a several fold increase in the incidence of NAS (1, 2). Using National Inpatient Sample (NIS) data (2004–2014), Winkelman et al. reported that one infant with NAS was born every 15 minutes. Medicaid financed births related to NAS contributed $462 million in hospital costs (3). Average national length of hospital stay for opioid exposed neonates is reported to be 16 days and prolonged hospitalization significantly adds to health care costs (3–5).

Timely and accurate prediction of NAS severity remains elusive secondary to the highly variable clinical expression in terms of onset, severity, and duration of signs (6–8). More accurate risk stratification for receipt of pharmacotherapy (PT) for NAS at the time of birth has several potential advantages. First, evidence-based resources could specifically be targeted for neonates at highest risk of severe NAS. A potential approach would be starting low dose PT such as Morphine prior to elevations in Finnegan Neonatal Abstinence Scoring System (FNASS) scores or abnormal Eat, Sleep, and Console (ESC) assessments (9). Accurate risk stratification will help inform therapeutic strategies that aim to minimize exposure while ensuring symptom control, aligning with current clinical guidelines. Next, accurate risk stratification can inform shared decision making with parents about expected disease trajectory, potential interventions and options for supportive care vs. pharmacologic therapy. Finally, risk stratification allows identification of patient population for clinical trials evaluating novel therapeutics e.g., drugs such as Clonidine and non-pharmacologic tools such as vibration mattresses or digital tools for care giver support.

Several predictive tools have been proposed to inform clinical decision making for treatment of NAS (10, 11). However, these tools have yet to be adopted in routine clinical practice primarily due to a lack of objective assessment of NAS, paucity of external validation and generalizability, and heterogeneity in the number and type of variables used in various models. Isemann et al. developed a prediction tool based on three specific signs of withdrawal (with or without opioid exposure category) assessed at 36 h of life to identify infants at risk for requiring PT for NAS, achieving high positive predictive values in a small, single-center retrospective cohort (*N* = 264) (10). However, the tool's reliance on subjective Finnegan scores and its postnatal timing limit its utility for early risk stratification at birth and reduce generalizability across diverse clinical settings. A recent retrospective study by Singh et al. analyzed a statewide database of over 2,000 opioid-exposed neonates to identify maternal, neonatal, and care-related factors associated with receipt of pharmacologic therapy for NAS and concluded that male sex, *in utero* exposure to medication treatment for maternal OUD with addition of non-prescription opioids, nicotine, benzodiazepines, SSRIs, maternal ineligibility to provide breast milk and out born infants were associated with higher likelihood of receipt of PT whereas skin to skin care and rooming in was associated with lower odds. While the study identified several important associations, its reliance on administrative data limits its utility for individualized risk stratification at birth. In the present study, pooled patient level data from two randomized control trials (RCTs) and three observational cohorts were used to derive a clinical predictive model to stratify opioid exposed neonates into two distinct risk groups (low, high) based on receipt of pharmacotherapy.

## Methods

### Data source and study cohorts

Data were pooled from two RCTs, two prospective observational research studies, and a retrospective community hospital cohort. Cohort size ranged from 79 to 392 neonates with the pooled cohort consisting of 698 infants. Inclusion and exclusion criteria for all cohorts are outlined below.

### Tufts Medical Center (*N* = 392)

This cohort included prospective data from an eight-site RCT representing northeast and southeast US (Massachusetts, Pennsylvania, Rhode Island, Maine, Florida and Tennessee) that compared methadone with morphine for the treatment of NAS (12) and a concurrent observational study of neonates whose parents consented for participation in the clinical trial but did not require treatment or whose parents refused consent for randomization in the clinical trial but consented to data collection (identical inclusion and exclusion criteria). Neonates were eligible for inclusion if their mothers received opioid agonist treatment for OUD during pregnancy with Methadone or Buprenorphine or received an opioid prescription for chronic pain. Neonates ≥37 weeks gestation with maternal history of psychotropic drug use for a known psychiatric diagnosis or illicit drug use during pregnancy were also included. Exclusion criteria included prenatal exposure to significant alcohol use, evidence of sepsis, major congenital anomalies, or genetic disorders.

### Cape Cod/Falmouth hospitals retrospective cohort (*N* = 79)

This cohort had retrospective data from two community hospitals in Cape Cod Massachusetts and had similar inclusion and exclusion criteria as the Tufts Medical Center trial (13).

### Thomas Jefferson University (*N* = 227)

This cohort was composed of eligible participants in a single center clinical trial of sublingual buprenorphine for treatment of NAS as well as a prospective observational study at the same center that enrolled all neonates at-risk for NAS based upon a history of *in utero* opioid exposure (14). The trial included neonates ≥37 weeks gestation exposed to opioids *in utero* and excluded infants with major congenital malformation, birth weight <2,200 g, serious medical or neurologic illness, seizures, hypoglycemia requiring treatment with intravenous glucose, and hyperbilirubinemia (serum bilirubin level >20 mg/dl). Neonates with maternal exposure to benzodiazepines for more than 30 days prior to delivery were also excluded.

### Inclusion/exclusion criteria

Neonates ≥37 weeks gestation born to pregnant women with OUD during the current pregnancy were eligible. Preterm neonates (<37 weeks gestation) were excluded given their variable length of and response to *in utero* opioid exposure.

### Primary outcome and exposures

The primary outcome was the receipt of PT for NAS based on modified FNASS criteria used to assess severity of NAS in all cohorts. A score was assigned every 4 h and treatment was initiated for a single score of ≥12 or 2 (Tufts Medical Center) or 3 (Thomas Jefferson University) consecutive scores of ≥8. Key independent variables considered to predict the binary primary outcome of receipt of PT are shown in Table 1. Co-exposure was defined as exposure to any of the substances or drugs other than Buprenorphine and Methadone and was determined by maternal self-report, maternal toxicology screens, and neonatal toxicology screens. Exposure to opioids was limited to methadone or buprenorphine for treatment of OUD and illicit opioids. Prescription opioid exposure was reported in only 32 of 730 mother- neonatal dyads in the initial pooled data set.

**Table 1:** Key independent variables considered for inclusion in the prediction model.

Demographics	Gestational age
	Sex
Neonatal characteristics	Birth weight
	Any breast milk
Maternal characteristics	Maternal race^a^
	Cesarean section delivery^a^
	Type of opioid for treatment of maternal OUD
	Methadone
	Buprenorphine
Co-exposures	Heroin
	Cocaine
	Benzodiazepines
	SSRIs
	Antipsychotics
	Alcohol^a^
	Tobacco
	Amphetamines^a^
	Gabapentin^a^

^a^ Not included in final model.

SSRIs, Selective Serotonin Reuptake Inhibitors.

### Sample size

Our initial pooled data set had 730 neonates [Tufts Trial/Observational cohort 416; BBORN/TJU trial 121; Cape Cod Hospitals 87; Thomas Jefferson University (TJU) Observational cohort 106]. The current analytic data set with data on demographic and clinical variables as well as the primary outcome has a sample size of 698 (430 treated, 62%) after exclusion of the 32 (24 from Tufts Medical Center and 8 from Cape Cod Hospital) neonates with maternal exposure to prescription opioids. Of note, there were no neonates with prescription opioids exposure from the remaining two cohorts (BBORN/TJU trial and TJU observational cohorts). With 268 non-events (38%), our data set could evaluate up to 13 predictors in the model to avoid model overfitting, following the 20 events per variable guideline (15).

### Missing data

While data on infant characteristics was almost complete, there was missing data on some maternal exposures. Among the key independent variables of interest, data on heroin exposure was missing for 107 (15.3%), cocaine exposure was missing for 85 (12.2%), type of maternal treatment opioid was missing for 17 (2.4%), amphetamine was missing for 115 (16.5%) and alcohol was missing for 240 (34.4%). Data on gabapentin exposure was missing for more than 50% of subjects.

### Statistical analyses

#### Variable selection

Potential candidate variables for model building were selected based on expert opinion, clinical judgement and previously published data (Table 1). Correlation matrix was used to detect collinear relationship among variables. Variables with more than 50% missingness were excluded (e.g., Gabapentin). Variables that had data missing across an entire cohort were also excluded (e.g., maternal race, alcohol exposure). Mode of delivery (C section vs. Vaginal) was considered as a candidate predictor based on its inclusion as a standard demographic variable in published literature of NAS. However, we chose to exclude it from final model given lack of a plausible biologic explanation for mode of delivery to influence risk for PT. We performed univariate comparisons between infants who did and did not receive PT to explore crude associations. However, significant relationships based on *P* value of less than 0.05 were not used to guide variable selection, consistent with PROBAST recommendations (16).

Missing data on key independent variables was addressed using multiple imputation (17, 18). Data were retrieved from electronic medical records (EMR) with inconsistent documentation on exposures and were handled under the assumption of missing at random (MAR). Values for these missing variables were imputed 10 times to generate 10 complete datasets utilizing “MICE” (Multivariate Imputation by Chained Equations) package in R studio. For each missing baseline variable, a regression model was generated to model the distribution of the missing variable as a function of all available data. This preserved the underlying variability, and distributional relationships present in the underlying data. Variables used in subsequent analyses as well as the outcome variable in the imputation model included: receipt of PT, gestational age, birth weight (grams), sex, any breast milk, type of maternal opioid for treatment of OUD, and exposure to tobacco, heroin, cocaine, benzodiazepines, SSRIs and/or antipsychotic medications.

### Model derivation

The final model was derived using multivariable logistic regression and specified a backward stepwise variable selection procedure. *P* value criterion of 0.157 was used to exclude or include variables at each step of model building. This high *P* value threshold was intentionally chosen as this aligns with Akaike Information Criterion (AIC) based variable retention. This approach is well supported in predictive modeling literature and helps avoid underfitting (19). In predictive modeling, the goal is not to identify statistically significant associations *per se*, but to maximize predictive accuracy. Traditional thresholds like *p* < 0.05 are designed for hypothesis testing and can result in the premature exclusion of variables that may contribute meaningfully to model performance.

To enable variable selection while using multiple imputation, all 10 imputed datasets were “stacked” into a single large dataset. To account for multiple observations for each subject, each entry was weighted by (1-f)/M where f equals the average fraction of missing data across all variables used in the imputation models and M is the number of imputed data sets (10) (17, 18). This approach is well supported in literature and was selected over traditional Rubin's Rules based on the following considerations: While Rubin's Rules are well-suited for pooling estimates from multiply imputed datasets once a final model is chosen, they are not easily applicable during the variable selection phase. When variable selection is performed separately within each imputed dataset, it often results in different sets of selected predictors across imputations. This inconsistency poses challenges for inference and model interpretability. Stacking allows us to leverage the full variability and sample size inherent in multiple imputation, thereby improving model stability and efficiency. The applied weights correct for pseudo-replication of observations across the imputed datasets. Wood et al. and Austin et al. highlight the trade-offs between different selection strategies and show that stacked datasets with weighting can achieve comparable performance to Rubin's Rules post-selection (17, 18). The 11 predictors included in the model building procedure were identical to those described above.

### Model validation

Due to lack of an independent cohort for external validation, the model was internally validated using bootstrap validation (20, 21). We utilized “boot_MI” function in “psfmi” package (R studio) (21) which bootstraps from the incomplete data set and applies multiple imputation in each boot strap sample. Five hundred bootstrap samples were generated from the original dataset and multiple imputation was used to generate 10 datasets for each bootstrap sample. Internal validation was conducted with backward variable selection for each bootstrap sample (including all candidate variables). Estimated slope value was used as a shrinkage factor to prevent our model from being overfitted in new data. This was done by multiplying the pooled coefficients with the shrinkage factor to determine a new intercept value aligned with the shrunken coefficients.

Leave-one-out validation (internal—external validation) (22) was then performed using data from two study cohorts to develop the model and conduct validation on the third cohort. This procedure was run for three unique combinations of cohorts, allowing us to examine stability of validation while also performing external validation.

### Model performance

Model performance was evaluated by measuring discrimination and calibration in each of the three cohorts. Model discrimination was determined by examining the area under the receiver operating characteristic curve (AUROC) and calibration assessed graphically by plotting observed risk of PT against deciles of predicted risk. Shrinkage factor was applied to adjust for optimism. A percent change in discrimination after adjusting for optimism was calculated using [(ValidationC-statistic−0.5)−(Derivation C-statistic−0.5)]/(DerivationC-statistic−0.5)×100](23). All statistical analyses were performed using R software, version 4.0.5 (R foundation for statistical computing, Vienna, Austria).

## Results

### Maternal and neonatal characteristics

A total of 698 infants were included in the model development with 61.6% receiving PT. None of the covariates were found to have a strong collinear relationship. Univariate comparisons were made between the 11 candidate predictors and receipt of PT with results provided in Table 2. Neonates who received PT were more likely to have been exposed to maternal treatment with methadone compared to buprenorphine (69 vs. 31%; *P*: 0.002) and were less likely to have received breast milk (46 vs. 63%; *P* = <0.001). There were no significant differences in demographic characteristics between the groups. Distribution of predictor variables across the cohorts is shown in Table 3.

**Table 2:** Maternal and neonatal characteristics and co-exposures by receipt of pharmacotherapy.

Characteristics	No PT	PT	*P* value	Missing *N* (%)
	(*N* = 268)	(*N* = 430)		
Neonatal characteristics				
Gestational age (weeks)	39.1 (1.2)	39.3 (1.2)	0.09	0 (0.0)
Female sex	133 (49.6)	223 (51.9)	0.62	0 (0.0)
Birth weight (grams)	3,092.1 (482.7)	3,105 (470.5)	0.73	0 (0.0)
Breast milk exposure	166 (63.1)	193 (46.3)	<0.001	18 (2.6)
Maternal characteristics				
White race	147 (93.6)	262 (89.7)	0.22	249 (35.6)
Treatment of OUD:			0.002	17 (2.4)
Methadone	147 (57.2)	293 (69.1)		
Buprenorphine	110 (42.8)	131 (30.9)		
C-section	69 (31.7)	128 (34.6)	0.52	110 (15.8)
Co-exposures				
Heroin	39 (17.3)	105 (28.8)	0.002	107 (15.3)
Cocaine	11 (4.7)	44 (11.5)	0.007	85 (12.2)
Benzodiazepines	9 (3.4)	65 (15.6)	<0.001	17 (2.4)
SSRIs	28 (10.5)	64 (15.1)	0.11	6 (0.9)
Antipsychotics	13 (4.9)	42 (9.8)	0.03	5 (0.7)
Alcohol	8 (5.0)	21 (7.1)	0.50	240 (34.4)
Tobacco	201 (75.6)	349 (81.9)	0.05	6 (0.9)
Amphetamines	5 (2.3)	9 (2.5)	1.0	115 (16.5)

Categorical variables expressed as frequencies and %, continuous variables as mean ± SD.

**Table 3:** Maternal, neonatal characteristics and co-exposures by study cohort.

Characteristics	Overall (*n* = 698)	Tufts (*n* = 392)	TJU (*n* = 227)	CCH (*n* = 79)
Neonatal characteristics				
Receipt of Pharmacotherapy	430 (61.6)	262 (66.8)	123 (54.2)	45 (57.0)
GA (weeks)	39.2 (1.24)	39.3 (1.3)	38.9 (1.2)	39.6 (1.3)
Female sex	356 (51)	206 (52.6)	111 (48.9)	39 (49.4)
Birth weight (g)	3,100.1 (474.9)	3,139.3 (503.4)	2,985.2 (421.7)	3,235.4 (406.2)
Breast milk exposure	359 (52.8)	226 (57.7)	91 (43.5)	42 (53.2)
Maternal characteristics				
White race	409 (91.1)	345 (90.3)	NA	64 (95.5)
Maternal treatment for OUD:	440 (64.6)	196 (50.0)	212 (98.6)	32 (43.2)
Methadone	241 (35.4)	196 (50.0)	3 (1.4)	42 (56.7)
Buprenorphine				
C- section delivery	197 (33.5)	139 (35.5)	29 (24.2)	29 (38.2)
Co-exposures				
Heroin	144 (24.4)	75 (19.4)	53 (40.2)	16 (22.2)
Cocaine	55 (9.0)	41 (10.7)	9 (5.9)	5 (6.6)
Benzodiazepines	74 (10.9)	57 (15.0)	11 (4.9)	6 (8.0)
SSRIs	92 (13.3)	50 (12.9)	33 (14.5)	9 (11.7)
Antipsychotics	55 (7.9)	35 (8.9)	16 (7.1)	4 (5.3)
Alcohol	29 (6.3)	23 (6.0)	NA	6 (8.2)
Tobacco	550 (79.5)	301 (77)	187 (83.9)	62 (79.5)
Amphetamines	14 (2.4)	8 (2.0)	4 (3.3)	2 (2.6)

Categorical variables—frequencies and %; continuous variables—mean ± SD.

### Receipt of pharmacotherapy

Table 4 displays the results of the final model which was derived from all study cohorts and yielded seven predictors of receipt of PT: gestational age, any breast milk, type of maternal treatment for OUD, and exposure to heroin, cocaine, benzodiazepines, and/or antipsychotic medications. All the predictor variables in the final model were associated with higher odds of receiving PT except for breast milk exposure. Exposure to methadone was associated with higher odds of receiving PT compared to buprenorphine (aOR: 1.57).

**Table 4:** Multivariable logistic regression model.

Characteristics	Estimate	OR	95% CI	*P*-value
Intercept	−8.04			
Neonatal characteristics				
Gestational age (weeks)	0.21	1.23	1.08–1.41	0.003
Breast milk exposure	−0.58	0.56	0.39–0.79	<0.001
Maternal characteristics				
Treatment of OUD:				
Buprenorphine		Ref		
Methadone	0.45	1.57	1.10–2.24	0.01
Co-exposures				
Heroin	0.43	1.53	1.00–2.34	0.05
Cocaine	0.56	1.75	0.82–3.73	0.15
Benzodiazepines	1.59	4.89	2.34–10.21	<0.001
Antipsychotics	0.66	1.94	0.97–3.85	0.06

C-statistic 0.68 (95% CI 0.64–0.72).

OR—adjusted odds ratios; CI, confidence intervals.

### Model performance

The final model derived using data from all three cohorts had an AUROC of 0.68 (95% CI: 0.64–0.72; optimism corrected 0.65 via bootstrapping). This decrement in discrimination from 0.68 to 0.65 reflects a percent change of approximately 17% calculated as [(ValidationC-statistic−0.5)−(DerivationC-statistic−0.5)]/ (DerivationC-statistic−0.5)×100] (23) A C- statistic of 0.7–0.8 is generally considered acceptable and 0.8–0.9 considered excellent (24, 25).

Although the model derived from the combination of Tufts and CCH cohorts achieved better discrimination with an AUROC of 0.73 (Table 5), it did not perform as well on external validation in TJU cohort (AUROC 0.65). The rest of the derivation cohorts had a C-statistic similar to the final model (Table 5).

**Table 5:** Model training and validation results.

Training			Validation		
Cohorts	AUROC	95% CI	Cohorts	AUROC	95% CI
Tufts, CCH	0.73	0.64–0.78	TJU	0.65	0.56–0.72
TJU, CCH	0.67	0.60–0.73	Tufts	0.67	0.61–0.72
TJU, Tufts	0.68	0.64–0.73	CCH	0.64	0.51–0.76

Tufts, TJU, CCH are the three study cohorts. TJU, Thomas Jefferson University; CCH, Cape Cod Health.

**Figure 1:**
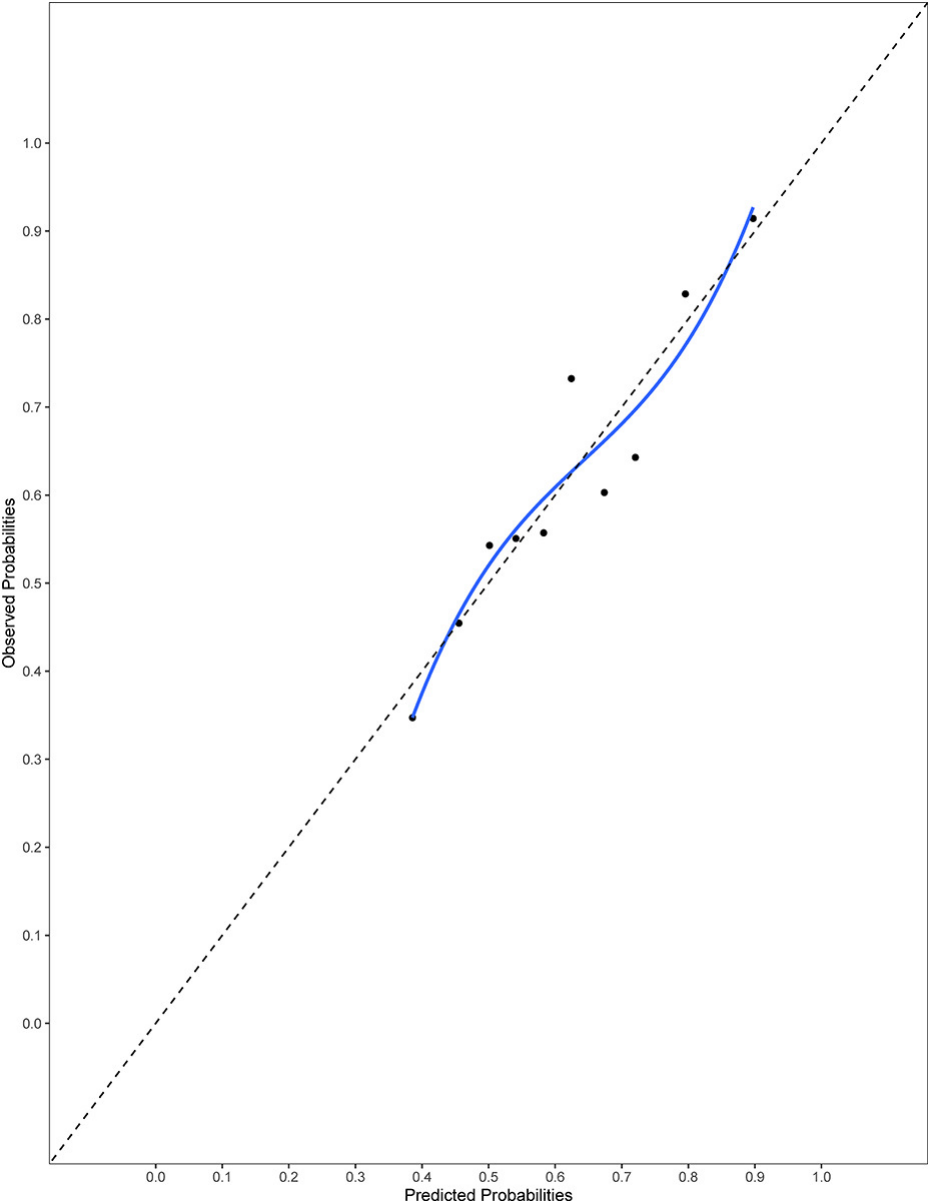
Calibration plot for the final model. Calibration slope 0.84 based on boot strap validation.

**Figure A2:**
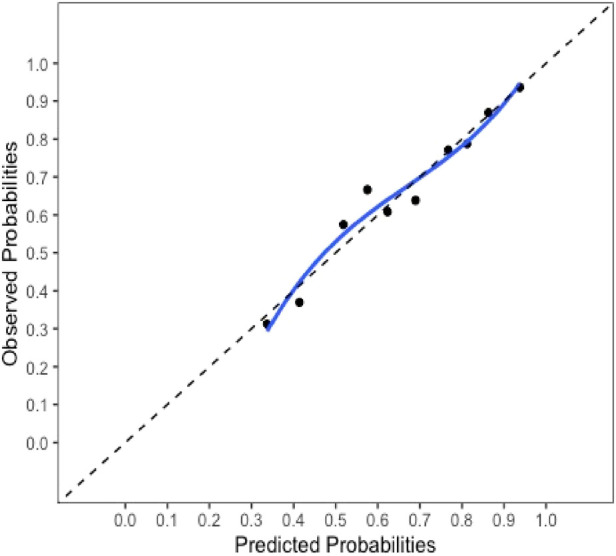
Calibration plot for model 1 derived from tufts and cape Cod cohorts.

**Figure A3:**
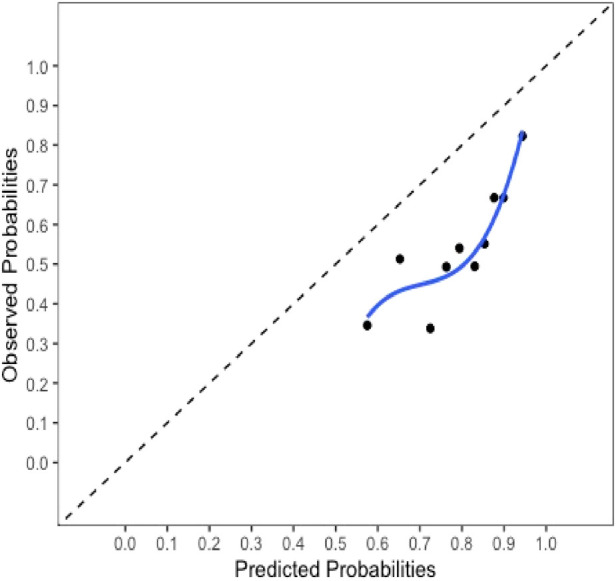
External validation of model 1 in TJU cohort.

**Figure A4:**
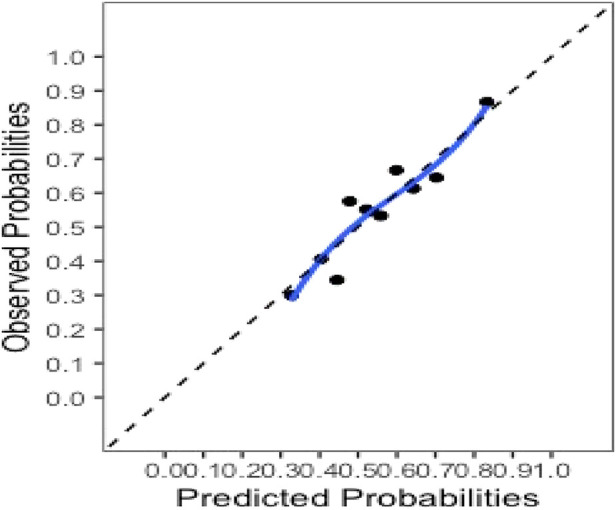
Calibration plot for model 2 derived from TJU and cape Cod cohorts.

**Figure A5:**
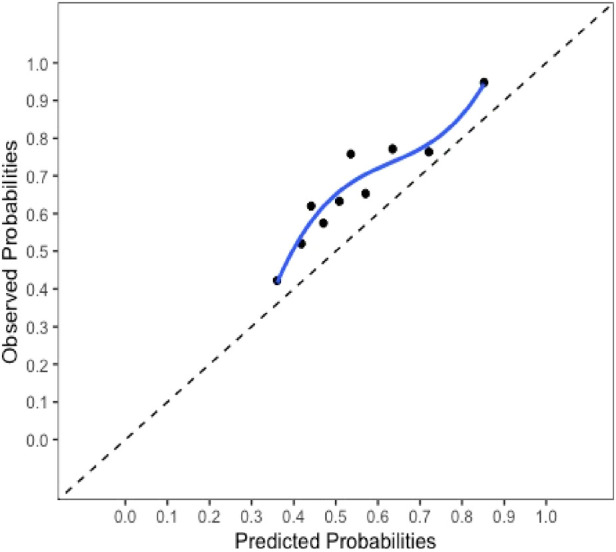
External validation of model 2 in tufts cohort.

**Figure A6:**
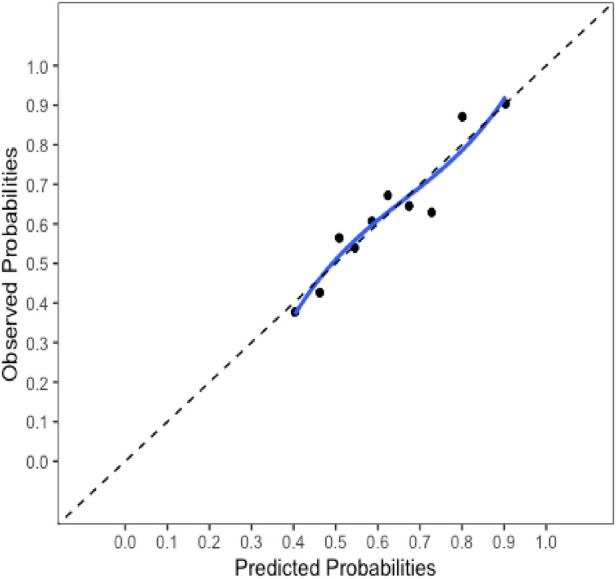
Calibration plot for model 3 derived from tufts and TJU cohorts.

**Figure A7:**
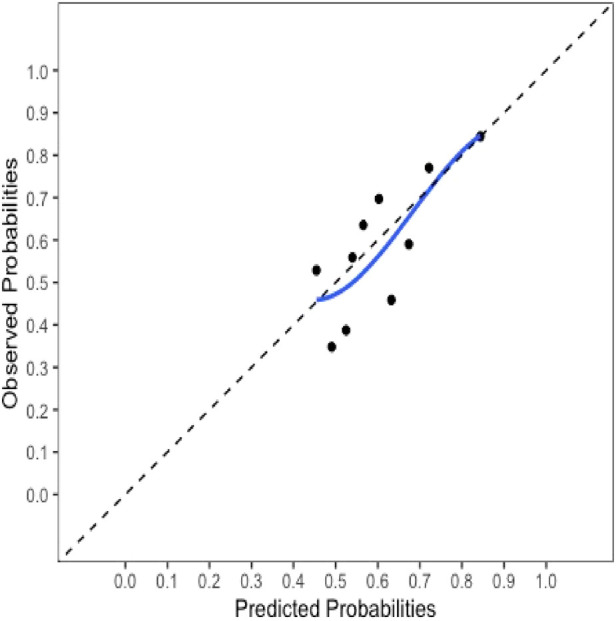
External validation of model 3 in cape Cod cohort.

The final model and the three training models appeared to calibrate well (Figure 1). However, models did not perform well within the external validation cohorts except the one derived from the combination of TJU and Tufts (Appendix Figures A2–A7). Calibration slope for the final model was 0.84 based on boot strap validation reflecting some overfitting.

## Discussion

Our multicenter, pooled cohort observational study identified seven specific maternal and neonatal clinical variables associated with NAS severity and receipt of PT. In neonates, increasing gestational age significantly increased the odds of receiving PT while breast milk significantly decreased the odds. Currently, the relationship between gestational age and NAS severity is not clearly understood (26, 27). Neonates with a higher gestational age have had a longer overall duration of exposure to *in utero* opioids with more mature opioid receptors, potentially increasing the level of physical dependence. Additionally, term infants likely have more rapid renal and hepatic clearance of the circulating opioids, which could potentiate the severity of NAS. Maternal breast milk exposure has been associated with less severe NAS. In a retrospective analysis of a statewide database of opioid exposed neonates, the lack of breastmilk was associated with higher odds of PT (11). Maternal breast milk is not only better tolerated but may also have trace amounts of maternal medications, both of which can help reduce NAS severity. Provision of breast milk is a key component of non-pharmacologic care practices along with skin-to-skin care, rooming in, and swaddling/holding the neonate which is well recognized to reduce the severity of NAS. Provision of maternal breast milk can be highly variable based on institutional guidelines and specific eligibility criteria, especially if illicit drug exposure is confirmed on toxicology screening.

There was no significant association between sex and receipt of PT in our model. This is in contrast to a recently published study that reported an association of receipt of PT with male sex (11). Another large population-based cohort study also demonstrated that males were more likely to develop NAS requiring PT (28). However, the study did not demonstrate sex-based differences in severity of NAS as evidenced by length of hospital stay (28). Sex dependent differences in salivary gene expression of neonates with NAS requiring PT has been reported (29). Clearly further research is warranted to explore the association between sex and severity of NAS.

Maternal treatment with Methadone was associated with higher odds of more severe NAS which is consistent with existing literature and reinforces the accuracy of our model (11, 30, 31). Other notable maternal exposures that were associated with higher odds of PT in the final model were exposure to heroin, benzodiazepines, and antipsychotic agents. Co -exposure to other psychotropic substances in addition to opioids is well recognized to contribute to severe NAS (e.g., prolonged therapy and increased use of second line medications) (32, 33). A model designed to predict the need for PT in NAS developed by Isemann et al. included four categories of exposures: (1) Buprenorphine, (2) Methadone, (3) Opioids other than Buprenorphine and Methadone, (4) Polysubstance exposure. Only polysubstance exposure was noted to be significantly associated with the need for PT (10). A notable exclusion in the final model was SSRI exposure. SSRIs are widely prescribed in pregnant persons with anxiety and depression and their use has been associated with increased severity of NAS in a clinical predictive model developed by Singh et al (11). In another recent study, Bakhireva et al. found that neonates co- exposed to maternal opioids and SSRIs were more than three times more likely to receive PT than those exposed to opioids alone (34). The lack of this association in our model could be due to the small number of subjects exposed to maternal SSRIs as well as lack of dosing data for this class of medication. Potential mechanisms include drug-drug interactions and direct neurobehavioral alterations independent from opioid withdrawal that can increase NAS severity scores (designed specifically for opioid exposure). Additionally, as highlighted by Lester BM et al., the association of neonatal withdrawal severity and PT with SSRIs co-exposure can represent an “artificial” inflation in neonatal withdrawal severity scores by virtue of having an “additive” effect on withdrawal signs from opioids (35). In order to have a better understanding of the true association of individual psychotropic agents with NAS treatment, more research is needed with adequately powered, well-designed studies. While addressing maternal mental health during pregnancy is critical, caution should be exercised when prescribing multiple psychotropic agents to pregnant individuals with OUD. Given the current gaps regarding safety and efficacy of these drugs (with the potential for drug-drug interactions), it is important to develop best practices based on more definitive research to guide healthcare providers in making informed treatment decisions that balance maternal mental health needs with neonatal outcomes. Further research is required to establish clearer guidelines for the safe and effective use of these medications during pregnancy.

Finally, while our findings demonstrate that co-exposure to certain psychotropic agents (e.g., benzodiazepines, antipsychotics) is associated with increased odds of PT in NAS, our study was not designed to directly evaluate pharmacokinetic or pharmacodynamic drug–drug interactions. These associations may reflect additive effects on neonatal withdrawal severity or confounding by underlying maternal psychiatric illness severity. Further research is needed to elucidate the mechanistic basis for these observed associations, including potential pharmacologic interactions.

Overall, our model is parsimonious (utilizing seven predictors) and discrimination was broadly consistent across the three derivation cohorts (AUROC 0.67–0.73) with only one model (Tufts, Cape Cod Hospitals) reaching threshold of good discrimination. It is also the first study to utilize geographically diverse multicenter patient level data to predict the receipt of PT in opioid exposed neonates. Early predictive tools developed by Isemann and colleagues included 21 signs of withdrawal from the Finnegan Scoring Tool as predictors of PT “within 36 h of birth” as well as some exposure data (10). Our study was unique in developing a model to predict the need for PT “at the time of birth” utilizing available demographic and exposure data.

### Limitations

While the internal and leave-one-out validation enhanced the methodological rigor of the study, several important limitations exist. All the included studies in the data set utilized FNASS for assessment of opioid exposed infants. While it would have been ideal to have both FNASS and ESC scoring tools in our dataset, this is a limitation that we acknowledge. However, the centers using ESC will still benefit from the results of this study as it helps identify infants at risk for more severe withdrawal at birth. A recent publication noted that the ability to diagnose and treat severe NAS is similar for the two approaches (FNASS and ESC) for monitoring and mainly impacted by other factors (36). The percentage of infants requiring pharmacotherapy in ESC approach varies considerably across institutions (37, 38). The only and largest trial to date that has directly compared ESC approach to usual care including the use of the Finnegan tool for NOWS reported about 19.5% use of PT in ESC group (37). In a retrospective review of medical records from a regional referral center in central Appalachia, 27% of infants required PT in the ESC period vs. 34% in pre-ESC period (*p* = 0.36) (38). These figures demonstrate that a significant proportion of infants still require pharmacotherapy with use of ESC. Clinical predictive models such as ours that aim to risk stratify infants will still have utility in settings using both FNASS or ESC approach as it helps: 1) guide conversations with parents and caregivers, 2) reallocate resources to high-risk infants (nurses, volunteer/cuddlers) and 3) implement non-pharmacological care modalities such as vibrating mattresses, noise reducing devices etc.

In our model there is lack of data on some clinically important variables that resulted in their exclusion and could have potentially contributed to a relatively modest discrimination. Variables of interest in this regard included exposure to gabapentin and maternal race which were either missing entirely across cohorts or had significant amount of missingness. Gabapentin is increasingly being prescribed to pregnant persons and co-exposure with opioids may be associated with an atypical or severe withdrawal syndrome in neonates (39). Data on alcohol use was also missing for one entire cohort and was excluded. Another notable exclusion was exposure to amphetamines which may be more common and relevant in some geographic locations. The missing data for included predictors ranged from 2.4% to 16.5% and was addressed using statistical methods (multiple imputation MI) that are well described in statistical literature. For data missing at random (MAR), simulation studies have shown that valid MI reduces bias even when the proportion of missing data is large (40). In our study we chose to not include variables with more than 50% missingness. The only variable of interest that fit this criterion was exposure to Gabapentin. Additionally, lack of accurate means of measurement of certain exposures could have compounded the predictive accuracy (e.g., alcohol use which is dependent on self-report and not routinely detected on toxicology screens). The internal-external validation demonstrated poor calibration within the external validation cohorts except the model derived from Tufts and TJU cohorts which was externally validated using the Cape Cod Hospitals cohort. Poor calibration likely reflects overfitting within derivation cohorts or could be attributed to unaddressed differences in eligibility criteria and outcome rates across the study cohorts. Specific data on the routine use of non-pharmacologic measures was not available, which has now been shown to be associated with improved outcomes.

There was fair heterogeneity across the study as demonstrated by variation in outcome frequency (54%–67%). This reflects variability in criteria used to initiate PT across the study cohorts, despite all sites utilizing a modified Finnegan NAS Scoring System. While some cohorts initiated treatment for a single FNASS score ≥12, others required two or three consecutive scores ≥8. These differences likely introduced variation in outcome assignment that may have affected both model calibration and the strength of associations between predictors and outcome. This limits the inter-study comparability in terms of reported exposure rates and subsequent model performance and was not addressed during modeling. This heterogeneity also reflects the variation in real-world clinical practice and underscores the challenge of developing universally applicable predictive models. Future studies should aim to incorporate more standardized or objective criteria for defining PT initiation in NAS to improve consistency across sites and enhance model performance. Finally, our model has not been validated using a fully independent external validation cohort and may not be suitable for reliable risk prediction in a broader opioid exposed neonatal population. Despite these limitations, our study builds upon prior work by using a multicenter dataset, transparent variable selection and internal-external validation. Additionally, we provide detailed justification for candidate predictors and a reproducible model development approach.

Our study demonstrates that prediction of a neonate's risk of receiving PT for NAS remains a challenging task. While several demographic and clinical factors involving maternal exposures and neonatal characteristics have been strongly associated with the need for PT, their predictive power is not sufficient to enable risk prediction at an individual patient level. Nevertheless, these frequently highlighted predictors need to be further investigated. With substantial increases in polysubstance use (licit and illicit) among pregnant persons and unknown interactions among psychotropic agents (including opioids) in this patient population, it is difficult to understand what precise drug-drug interactions substantially enhance risk of NAS in an individual infant. Better understanding of the biologic pathways in which these drugs interact and are metabolized will help delineate exposures or combination of exposures that significantly increase risk of a neonate being treated for NAS. Furthermore, challenges in prediction are also magnified by the lack of a gold standard definition for diagnosing NAS across clinical as well as research settings (41). The majority of NAS definitions and diagnoses are linked to a neonate's scores on the modified versions of Finnegan NAS Scoring System or use of administrative coding data (42). These scoring tools are inherently subjective and greatly influenced by inter- rater variability. This variation in NAS definition across centers and studies is likely to impact predictive model performance in external validation by limiting inter-study comparability in event rates. In the future, addition of genomic data such as Polygenic Risk Scores (PRS) to metabolomic and proteomic biomarkers and comprehensive clinical and demographic data in larger cohorts of neonates could provide greater accuracy in identifying neonates at risk for developing more severe forms of NAS (43).

## Conclusion

There is an urgent need to develop objective clinical tools to accurately predict NAS severity to facilitate the optimal precision medicine approach for neonates born following *in- utero* opioid exposure. We have attempted to overcome the current limitations for establishing clinical utility of the existing predictive models such as validity, small sample size, data from a single center, or claims based with variation in coding for NAS. Future work should focus on establishing large and diverse NAS data registries and obtaining more definitive data on safety and efficacy of polypharmacy in this population. These efforts are currently underway through the Helping End Addiction Long term (HEAL) program supported by the National Institutes of Health in the US.

## Data Availability

The data analyzed in this study is subject to the following licenses/restrictions: deidentified data for this study was sourced from clinical trials conducted in the past. Deidentified data can be made available on request. Requests to access these datasets should be directed to Shawana Bibi MD, bibis@ccf.org.

## Appendix

This document contains calibration plots for the three training models and internal-external validation.
